# Evolution of a neuromuscular sexual dimorphism in the *Drosophila montium* species group

**DOI:** 10.1038/s41598-021-94722-3

**Published:** 2021-07-27

**Authors:** Han-qing Liang, Toru Katoh, Kosei Sato, Daisuke Yamamoto, Shuo-yang Wen

**Affiliations:** 1 Department of Entomology, College of Plant Protection, South China Agricultural University, Guangzhou, 510642 China; 2 Department of Biological Sciences, Faculty of Science, Hokkaido University, Sapporo, Hokkaido 060-0810 Japan; 3 Advanced ICT Research Institute, National Institute of Information and Communications Technology, Kobe, 651-2492 Japan

**Keywords:** Evolutionary genetics, Sexual selection, Entomology

## Abstract

While epigamic traits likely evolve via sexual selection, the mechanism whereby internal sexual dimorphism arises remains less well understood. Seeking clues as to how the internal sexual dimorphism evolved, we compared the abdominal musculature of 41 *Drosophila montium* group species, to determine whether any of these species carry a male-specific muscle of Lawrence (MOL). Our quantitative analysis revealed that the size of a sexually dimorphic MOL analog found in 19 *montium* group species varied widely from species to species, suggesting the gradual evolution of this sexually dimorphic neuromuscular trait. We attempted the ancestral state reconstitution for the presence or absence of the neuromuscular sexual dimorphism in the A5 segment; the neuromuscular sexual dimorphism existed in an old ancestor of the *montium* group, which was lost in some of the most recent common ancestors of derived lineages, and subsequently some species regained it. This loss-and-gain history was not shared by evolutionary changes in the courtship song pattern, even though both traits were commonly regulated by the master regulator male-determinant protein FruM. It is envisaged that different sets of FruM target genes may serve for shaping the song and MOL characteristics, respectively, and, as a consequence, each phenotypic trait underwent a distinct evolutionary path.

## Introduction

Sexually dimorphic characteristics have been a focus of evolutionary studies because they represent an important signature of the history of sexual selection^1^. Although male-specific external structures have attracted substantial interest among biologists^1,2^, sex differences in the structure and function of internal organs have not been thoroughly studied from an evolutionary point of view^3^. As an attempt to understand how internal sexual dimorphisms evolve, we studied a male-specific muscle called the muscle of Lawrence or MOL^4^ in the *Drosophila montium* group^5,6^, in which the male courtship display varies widely across species^7,8^, suggesting a unique evolutionary history of sexually dimorphic traits.

The MOL represents a single pair of longitudinal muscles running along the dorsal tergite of the fifth abdominal segment (A5) of adult males in some *Drosophila* species^9,10^. In *D. melanogaster*, where MOL formation has been studied in some depth, the MOL is readily recognizable as it is longer and wider than other longitudinal muscles. Gynandromorphic flies may or may not carry the MOL, depending on the sex chromosomal composition of the innervating motoneuron^11,12^, now known as the MOL-inducing (Mind) neuron^13^; the MOL forms when the composition is XO (the male) but not XX (the female), whereas the chromosomal sex of myocytes composing the muscle has nothing to do with the MOL formation^14–16^.Thus, the presence of MOL signifies that the innervating neuron is a male cell, whereas its absence indicates that the innervating neuron is a female cell. The MOL is larger than conventional longitudinal muscles as a result of a larger number of recruited myocytes, which fuse to form a fiber of the MOL.

Sex determination in *Drosophila* involves a cascade of genes that produce RNA splicing factors, the terminal effectors of which are two transcription factor genes, *doublesex* (*dsx*) and *fruitless* (*fru*). Expression of *fru* in the Mind neuron is essential for the MOL formation^17–20^, whereas *dsx* seems to play no role^21,22^. Indeed, female flies acquire the male-specific MOL if the male-specific *fru* gene product (i.e., the FruM protein) is transgenically produced in innervating motoneurons^20^.

FruM functions during the pupal stage as a master regulator of the formation of adult neural circuits underlying male mating behavior in *D. melanogaster*. In this species, ~ 2000 FruM-expressed neurons distributed through the entire nervous system from the sensory to the central and motor systems are interconnected, forming a circuit (i.e., the *fru* circuit) that operates to generate courtship behavior^23,24^. Although the MOL-innervating Mind neuron expresses FruM as aforementioned and thus likely contributes to the *fru* circuit, no role in courtship behavior has been assigned to the Mind-MOL neuromuscular system. Although the functional significance of the MOL remains obscure, this muscle offers an experimentally tractable system for the analysis of the *fru*-dependent developmental mechanism even in non-model species where the possibility of genetic manipulation of the *fru* circuit is limited. We therefore decided to use the MOL metrics as a convenient and reliable proxy of the FruM activity in the nervous system and compared them across species of the *montium* group to infer the evolutionary history of the *fru* circuit.

Although the *fru* circuit in non-*melanogaster* drosophilid flies is ill-defined due to technical difficulties in visualizing and manipulating specific neurons in these species, a few successful studies support the idea that the *fru* circuit plays a central role in executing courtship behavior also in these species^25,26^. Courtship song is a hallmark of *Drosophila* mating displays that are *fru*-dependent^27,28^ and *montium* group members exhibit spectacular variations in singing behavior^29,30^. Remarkably, males of many species of the *montium* group generate song not only before mounting (pre-mounting songs) but also after mounting (post-mounting songs), in contrast to most other *Drosophila* groups, in which males sing only pre-mounting songs^29,30^. Thoracic muscles on their own cannot produce any courtship song. They need to be driven by the FruM-dependent central neural circuit, a system distributed across the entire body, including the abdomen, in which the MOL and the FruM-positive innervating motoneurons exist. The interplay between the abdominal and thoracic circuitries is critical when a courting male makes an attempt at copulating, because the thoracic leg motor coordination is pivotal in bringing all his body parts into an appropriate position. Additionally, it is likely that males of some *montium* group species commence singing a post-mounting song when the thoracic song pattern generator becomes active upon receiving inputs from the abdominal ganglion that signals successful genital contact with a mating partner. These considerations suggest that the FruM-expressing neurons in the thorax and abdomen (and indeed the head) operate in coordination to generate courtship actions including singing, and therefore, it is logically possible that the MOL (as an abdominal muscle) and the song (powered by the thoracic muscles) evolved under common selective pressures.This might suggest that the *montium* group underwent substantial diversification in the neural *fru* activity across clades, prompting us to explore such evolutionary *fru* activity changes by comparing MOLs among the species of this group. This was our rationale for comparing the evolutionary history of the MOL formation with that of the song pattern.

A cross-species comparison of the MOL in the genus *Drosophila* (sensu lato) has been made by Gailey et al.^9^, who examined male flies of 95 species and classified them into two categories, i.e., species with the MOL (MOL+: 28 species) and those without the MOL (MOL−: 67 species), based on visual inspection of the abdominal musculature without quantification of the muscle size. That study included 12 species from the *montium* group, all of which were judged to be MOL−. In view of the fact that *fru*-dependent song traits are highly variable from species to species in the *montium* group, one may anticipate that the MOL formation, another *fru*-dependent trait, would also have been diversified, contrary to the observation by Gailey et al.^9^.

Through quantitative comparisons of the muscle size by standardized image analysis and statistical tests, here we show that some of the *montium* group species carry a single pair of sexually dimorphic muscles in the A5 abdominal segment, reminiscent of the MOL in *D. melanogaster*. These sexually dimorphic muscles in the *montium* group species are, however, much smaller in size than the MOL in *D. melanogaster* and exhibit substantial variations in size among species. We postulate that these sexually dimorphic muscles in the *montium* groups species represent the MOL analogs and suggest that the formation of MOL and its analogs evolved gradually, rather than emerging in an all-or-none manner across lineages. However, no apparent correlation was found between the putative MOL analogs and song characteristics in the *montium* group species, implying that these two traits evolved under distinct selective pressures.

## Results

### MOL quantification in the outgroup species

**Figure 1:**
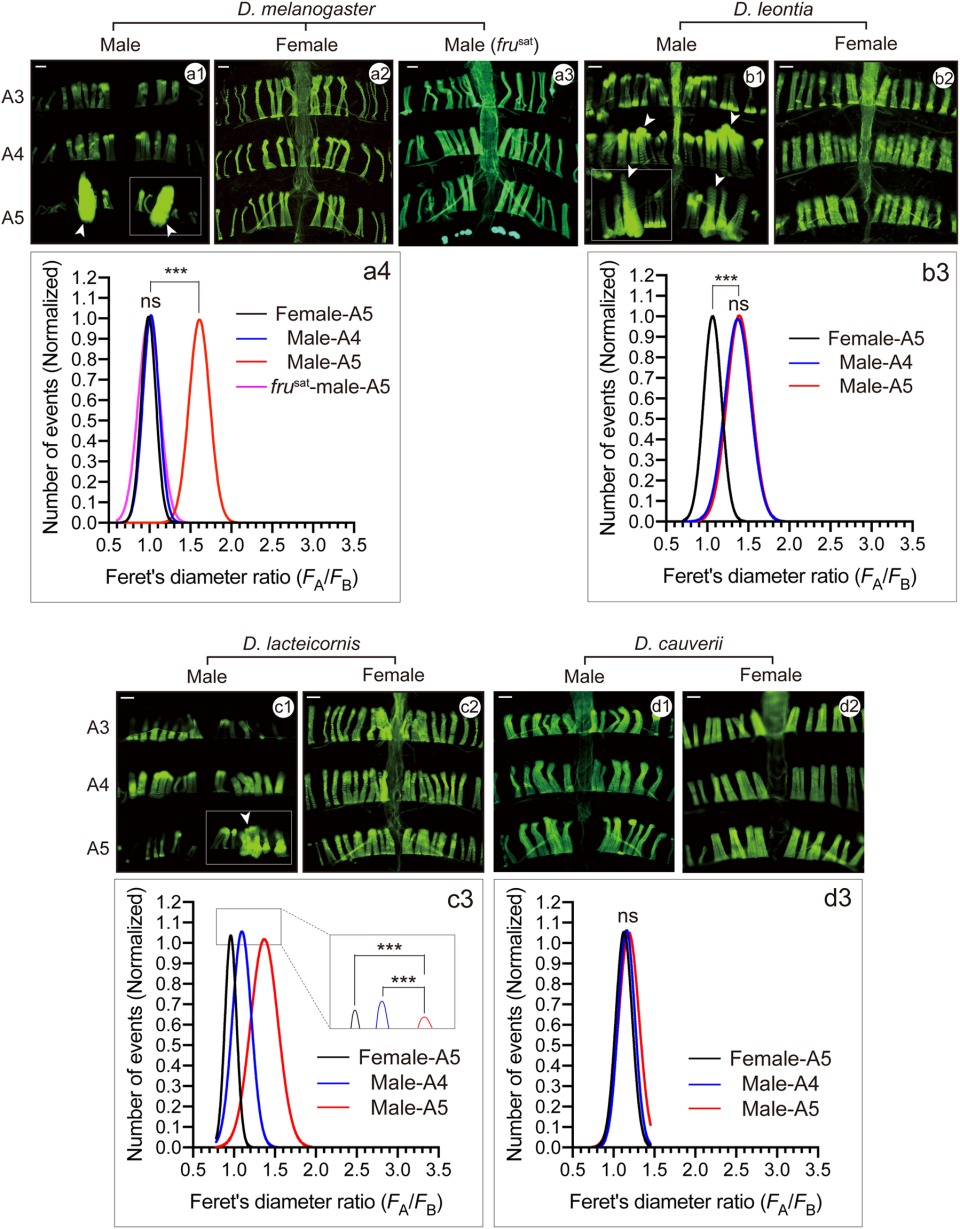
Detection of sexual dimorphism in the abdominal musculature of *D. melanogaster* and some species of the *montium* group. Representative images (**a1**–**a3**,**b1,b2,c1,c2**,**d1,d2**) of abdominal muscles and the Gaussian fit to the *F*_*A*_*/F*_*B*_ distribution of the largest muscle (**a4**,**b3,c3**,**d3**) in the reference species *D. melanogaster* (**a**) and 3 species of the *montium* groups, i.e., *leontia* (**b**), *lactericornis* (**c**) and *cauverii* (**d**). Images are typical examples of male abdominal musculatures with the exception of those for *D. melanogaster*, which are images of a wild-type male (**a1**), wild-type female (**a2**) and *fru* mutant male (**a3**). The MOL and its analogs are indicated with arrowheads, and the regions shown in Fig. 4 to visualize nuclei are boxed. *D. cauverii* is a species that did not show the neuromuscular sexual dimorphism in the A5 segment whereas 3 other species displayed the sexual dimorphism. Scale bar: 100 µm. The curves in **a4**, **b3**, **c3** and **d3** compare *F*_*A*_*/F*_*B*_ distributions for the male A5 (red lines), the female (black lines) and male A4 (blue lines), and, only in **a4**, the male *fru* mutant A5 (magenta lines). The statistical differences were evaluated by the Brown-Forsythe and Welch ANOVA or Kruskal–Wallis test; ***P < 0.001.

We first characterized the MOL and candidate MOL analogs in the following seven outgroup species: *D. melanogaster, D. erecta, D. yakuba, D. subobscura, D. affinis, D. virilis* and *D. mercatorum* (Table S1). In *D. melanogaster* males, the MOL is readily recognizable because of its large size and unique positioning in the A5 segment (Fig. 1a1; cf. Fig. 1a2). The length of the MOL also exceeds that of conventional muscles in *D. subobscura*, whose MOL has been extensively characterized because of its unusual feature, i.e., the presence of an additional pair of MOLs in A4^9,26^. We therefore decided to use the length difference in distinguishing the MOL from conventional muscles. As a rigorous means to define the length, we adopted Feret’s diameter as calculated by the Fiji package, an ImageJ standard option (https://imagej.net/Welcome: see Materials and Methods; Fig. S1). Feret’s diameter was measured for the putative MOL (*F*_*A*_) and for the medial-most muscle running along the midline, which served as a control (*F*_*B*_) within the same hemi-segment for each fly. The frequency histogram of *F*_*A*_*/F*_*B*_ constructed for the longest muscles in male A5 in *D. melanogaster* was fitted with a Gaussian distribution function, yielding the mean ± SEM of 1.71 ± 0.03 (Fig. 1a). Similar plots were constructed for the muscles in the male A4 and female A5 segments of the wild type and also the *fru* mutant male A5 in *D. melanogaster*, giving *F*_*A*_*/F*_*B*_ values of 1.00 ± 0.01, 0.99 ± 0.01 and 1.00 ± 0.02, respectively (Fig. 1a; Table S1). The longest muscles in male A5 were significantly longer than conventional muscles in male A4 or female A5 segments (Fig. 1a), supporting the notion that the former muscles represent the analogs of MOL, which is male-specific and A5-specific in *D. melanogaster*.

Similar analysis in *D. subobscura* (Fig. S2) revealed that the longest muscles in male A5 (*F*_*A*_*/F*_*B*_ = 1.93 ± 0.04) were significantly longer than conventional muscles in female A5 (1.12 ± 0.02) but not longer than the counterparts in male A4 (2.13 ± 0.04), an observation consistent with previous studies showing that *D. subobscura* males carry an additional pair of the MOL in A4^9,10,26^. We also constructed a frequency histogram of *F*_*A*_*/F*_*B*_ for the longest muscles in male A5, male A4 and female A5 in *D. erecta*, *D. yakuba*, *D. affinis, D. virilis* and *D. mercatorum* (Fig. S2). The histogram showed that the mean length of the longest muscles in male A5 (*F*_*A*_*/F*_*B*_ = 1.34 ± 0.02) and also male A4 (1.32 ± 0.02) was longer than that in female A5 (1.13 ± 0.01) in *D. affinis* (Fig. S2), despite a previous work reporting that MOL was absent in this species^9^. The MOL-like muscles were not found in the remaining four outgroup species (Table S1).

Therefore, the quantification of the MOL and its potential analogs in the seven outgroup species described above suggests that the estimates of the muscle length with Feret’s diameter are in good agreement with their relative sizes judged by visual inspection. We thus used Feret’s diameter *F*_*A*_*/F*_*B*_ to quantify the MOL-analog formation in the subsequent analysis with the *montium* group (Table 1).

**Table 1:** Feret's diameter of the largest muscle of abdominal segments in male and female flies of the *montium* group.

Subgroup/ (Group)	Species	Strain code	Female					Male									Statistical test used	Female-A5 versus Male-A5		Male-A4 versus Male-A5		MOL analog
			*N*	A5				*N*	A4					A5								
				*n*	*F*_A_/*F*_B_				*n*	*F*_A_/*F*_B_			*n*	*F*_A_/*F*_B_				Statistical significance	Multiplicity adjusted P value	Statistical significance	Multiplicity adjusted P value	
					Mean ± SEM	Min	Max			Mean ± SEM	Min	Max		Mean ± SEM	Min	Max						
*montium*	*D. asahinai*	AM06-12	34	68	1.09 ± 0.01	0.90	1.30	35	70	1.12 ± 0.02	0.72	1.53	70	1.14 ± 0.01	0.84	1.52	ANOVA	**	0.005	ns	0.62	−
	*D. auraria*	A662	36	72	1.06 ± 0.01	0.93	1.27	34	68	1.10 ± 0.01	0.94	1.50	68	1.07 ± 0.01	0.91	1.32	ANOVA	ns	0.72	ns	0.17	−
	*D. baimaii*	ML11023	36	72	1.04 ± 0.01	0.89	1.18	36	72	1.18 ± 0.02	0.91	1.45	72	1.38 ± 0.02	0.97	1.82	ANOVA	***	< 0.001	***	< 0.001	+
	*D. biauraria*	B16	35	68	1.02 ± 0.01	0.88	1.23	33	66	1.08 ± 0.01	0.79	1.33	66	1.08 ± 0.01	0.89	1.41	Kruskal–Wallis test	**	0.002	ns	> 0.99	−
	*D. fengkainensis*	XT33	33	66	1.02 ± 0.01	0.88	1.20	36	60	1.10 ± 0.01	0.97	1.30	60	1.08 ± 0.01	0.78	1.25	Kruskal–Wallis test	***	< 0.001	ns	> 0.99	+
	*D. lacteicornis*	IRUR20	41	82	0.97 ± 0.01	0.84	1.39	37	74	1.10 ± 0.01	0.78	1.39	74	1.38 ± 0.02	1.03	1.86	ANOVA	***	< 0.001	***	< 0.001	+
	*D. neoasahinai*	OKNH2K	33	66	1.00 ± 0.01	0.80	1.27	37	70	1.01 ± 0.02	0.74	1.50	70	1.08 ± 0.02	0.84	1.40	Kruskal–Wallis test	**	0.004	**	0.0022	−
	*D. pectinifera*	OGS98m	38	76	1.06 ± 0.01	0.84	1.25	40	80	1.13 ± 0.01	0.92	1.59	80	1.22 ± 0.01	0.85	1.48	ANOVA	***	< 0.001	***	< 0.001	+
	*D. pseudobaimaii*	ML11250	32	64	1.03 ± 0.01	0.80	1.21	34	68	1.13 ± 0.01	0.88	1.43	68	1.08 ± 0.01	0.84	1.40	ANOVA	**	0.002	*	0.02	−
	*D. rufa*	rufa-OGM	37	74	0.98 ± 0.01	0.82	1.26	37	72	1.00 ± 0.01	0.81	1.27	72	1.00 ± 0.01	0.86	1.36	ANOVA	ns	0.28	ns	0.93	−
	*D. subauraria*	ONM29	32	64	1.06 ± 0.01	0.83	1.20	36	72	1.08 ± 0.01	0.80	1.35	72	1.11 ± 0.01	0.91	1.33	ANOVA	**	0.003	ns	0.27	−
	*D. tani*	MES01	36	71	1.01 ± 0.01	0.86	1.25	41	79	1.01 ± 0.01	0.75	1.21	78	1.05 ± 0.01	0.85	1.46	Kruskal–Wallis test	*	0.04	ns	0.11	−
	*D. trapezifrons*	Bavi31	40	79	0.98 ± 0.01	0.85	1.12	39	78	1.14 ± 0.01	0.94	1.49	78	1.10 ± 0.01	0.91	1.46	ANOVA	***	< 0.001	*	0.04	+
	*D. triauraria*	T544	34	68	1.03 ± 0.01	0.78	1.30	34	68	1.11 ± 0.01	0.91	1.47	64	1.04 ± 0.01	0.81	1.28	ANOVA	ns	0.95	***	< 0.001	−
*kikkawai*	*D. bocki*	IR2-37	36	72	1.14 ± 0.01	0.97	1.47	37	74	1.26 ± 0.02	0.92	1.63	74	1.41 ± 0.02	1.05	1.92	ANOVA	***	< 0.001	***	< 0.001	+
	*D. kikkawai*	OGH06-01	37	74	1.07 ± 0.01	0.92	1.25	43	86	1.17 ± 0.01	0.75	1.47	86	1.51 ± 0.03	1.15	2.16	Kruskal–Wallis test	***	< 0.001	***	< 0.001	+
	*D. leontia*	AO-2	37	73	1.07 ± 0.01	0.88	1.40	44	88	1.36 ± 0.02	1.02	2.18	88	1.40 ± 0.02	1.06	1.82	ANOVA	***	< 0.001	ns	0.15	+
	*D. lini*	BGS3146.1	33	65	1.03 ± 0.01	0.79	1.33	32	64	1.04 ± 0.01	0.87	1.26	64	1.07 ± 0.01	0.92	1.31	ANOVA	**	0.009	ns	0.05	−
	*D. ogumai*	RGN3	39	77	1.00 ± 0.01	0.79	1.28	37	74	1.11 ± 0.02	0.83	1.62	73	1.23 ± 0.02	0.86	1.52	ANOVA	***	< 0.001	***	< 0.001	+
	*D. ohnishii*	ML45	36	72	1.05 ± 0.01	0.81	1.32	38	76	1.09 ± 0.01	0.81	1.43	76	1.26 ± 0.01	0.96	1.74	ANOVA	***	< 0.001	***	< 0.001	+
*punjabiensis*	*D. punjabiensis*	CJB212	35	70	1.06 ± 0.01	0.80	1.30	38	74	1.14 ± 0.01	0.85	1.42	74	1.11 ± 0.02	0.85	1.51	ANOVA	ns	0.07	ns	0.18	−
	*D. watanabei*	14028-0531.02	40	79	1.05 ± 0.01	0.86	1.28	39	77	1.17 ± 0.01	0.95	1.45	78	1.20 ± 0.01	0.84	1.40	ANOVA	***	< 0.001	ns	0.27	+
*orosa*	*D. orosa*	14028-0611.00	33	64	1.05 ± 0.01	0.87	1.31	34	68	1.08 ± 0.01	0.88	1.44	68	1.10 ± 0.02	0.91	1.67	ANOVA	*	0.01	ns	0.57	−
*serrata*	*D. barbarae*	ML11213	39	77	1.03 ± 0.01	0.85	1.24	36	71	1.23 ± 0.02	0.88	1.81	71	1.60 ± 0.03	1.21	2.22	ANOVA	***	< 0.001	***	< 0.001	+
	*D. bicornuta*	BOG1	11	22	1.09 ± 0.02	0.98	1.35	36	72	1.09 ± 0.01	0.87	1.44	72	1.08 ± 0.01	0.93	1.38	ANOVA	ns	0.85	ns	0.89	−
	*D. birchii*	14028-0521.00	39	77	1.02 ± 0.01	0.87	1.33	37	74	1.11 ± 0.01	0.83	1.47	74	1.15 ± 0.01	0.89	1.34	Kruskal–Wallis test	***	< 0.001	**	0.005	+
	*D. bunnanda*	14028-0721.00	33	66	1.21 ± 0.02	0.91	1.61	33	65	1.35 ± 0.02	1.02	1.73	66	1.28 ± 0.02	0.89	2.17	ANOVA	*	0.03	ns	0.08	−
	*D. cauverii*	cauv-CNRS	37	74	1.14 ± 0.01	0.89	1.40	38	76	1.15 ± 0.01	0.96	1.38	75	1.18 ± 0.01	0.84	1.39	ANOVA	ns	0.07	ns	0.26	−
	*D. mayri*	14028-0591.00	37	73	1.10 ± 0.01	0.86	1.62	36	71	1.22 ± 0.02	0.71	1.76	72	1.53 ± 0.02	1.17	2.02	ANOVA	***	< 0.001	***	< 0.001	+
	*D. serrata*	Q122	41	82	1.09 ± 0.01	0.85	1.29	37	72	1.18 ± 0.01	0.90	1.55	74	1.21 ± 0.02	0.95	1.94	ANOVA	***	< 0.001	ns	0.47	+
	*D. truncata*	RGN179	38	76	1.12 ± 0.01	0.93	1.35	36	72	1.05 ± 0.01	0.92	1.31	72	1.27 ± 0.01	0.99	1.55	ANOVA	***	< 0.001	***	< 0.001	+
*seguyi*	*D. burlai*	L6	34	68	1.07 ± 0.01	0.89	1.26	32	64	1.20 ± 0.02	0.96	1.46	64	1.13 ± 0.01	0.90	1.36	ANOVA	**	0.001	**	0.001	−
	*D. diplacantha*	dip05860	39	78	1.15 ± 0.01	0.97	1.70	40	74	1.22 ± 0.02	0.85	1.59	74	1.23 ± 0.01	0.85	1.51	ANOVA	***	< 0.001	ns	0.88	+
	*D. greeni*	14028-0712.00	35	70	1.05 ± 0.01	0.79	1.27	37	74	1.11 ± 0.01	0.79	1.37	73	1.14 ± 0.02	0.88	1.62	ANOVA	***	< 0.001	ns	0.31	+
	*D. jambulina*	F76	36	70	1.04 ± 0.01	0.84	1.25	36	72	1.00 ± 0.01	0.82	1.30	71	1.07 ± 0.01	0.88	1.26	ANOVA	ns	0.11	***	< 0.001	−
	*D. malagassya*	J6	37	73	1.10 ± 0.01	0.79	1.48	35	70	1.14 ± 0.02	0.86	1.47	70	1.07 ± 0.01	0.76	1.31	ANOVA	ns	0.17	*	0.01	−
	*D. nikananu*	14028-0601.00	37	73	1.01 ± 0.01	0.75	1.29	34	62	1.09 ± 0.02	0.68	1.46	62	1.07 ± 0.02	0.80	1.53	ANOVA	*	0.04	ns	0.72	−
	*D. seguyi*	K59	41	81	1.10 ± 0.01	0.87	1.39	37	73	1.14 ± 0.01	0.88	1.53	74	1.18 ± 0.02	0.88	1.64	ANOVA	**	0.002	ns	0.4	−
	*D. tsacasi*	14028-0701.00	33	65	1.06 ± 0.01	0.84	1.45	32	64	1.20 ± 0.02	0.87	1.58	64	1.17 ± 0.01	0.92	1.44	ANOVA	***	< 0.001	ns	0.46	+
	*D. vulcana*	14028-0711.00	38	75	1.07 ± 0.01	0.88	1.69	34	68	1.01 ± 0.01	0.83	1.18	68	1.07 ± 0.01	0.89	1.41	ANOVA	ns	0.96	***	< 0.001	−
*parvula*	*D. kanapiae*	14028-0541.00	35	69	1.11 ± 0.02	0.87	1.57	34	67	1.10 ± 0.01	0.92	1.37	67	1.15 ± 0.01	0.90	1.55	ANOVA	ns	0.14	**	0.01	−
	*D. parvula*	ML11218	34	68	1.21 ± 0.01	0.94	1.47	36	70	1.28 ± 0.02	1.00	1.62	70	1.23 ± 0.01	0.98	1.53	ANOVA	ns	0.54	ns	0.07	−

*N*: The total number of individuals used in this analysis. *n*: The total number of A4 or A5 hemi-segments examined. *F*: Feret's diameter (*F*_*A*_ and *F*_*B*_), the longest distance of a target muscle. *F*_*A*_/*F*_*B*_: The Feret's diameter ratio obtained by dividing the Feret’s diameter of the longest muscle (*F*_*A*_) by that of the medial-most conventional muscle (*F*_*B*_) in the same hemi-segment. When the mean *F*_*A*_/*F*_*B*_ in males is larger than that in females at the statistically significant level of *P* < 0.001, we judge that the males have the MOL analog or without MOL as indicated by a "+" or "−".

### Sex differences in MOL analogs of *montium* group species

**Figure 2:**
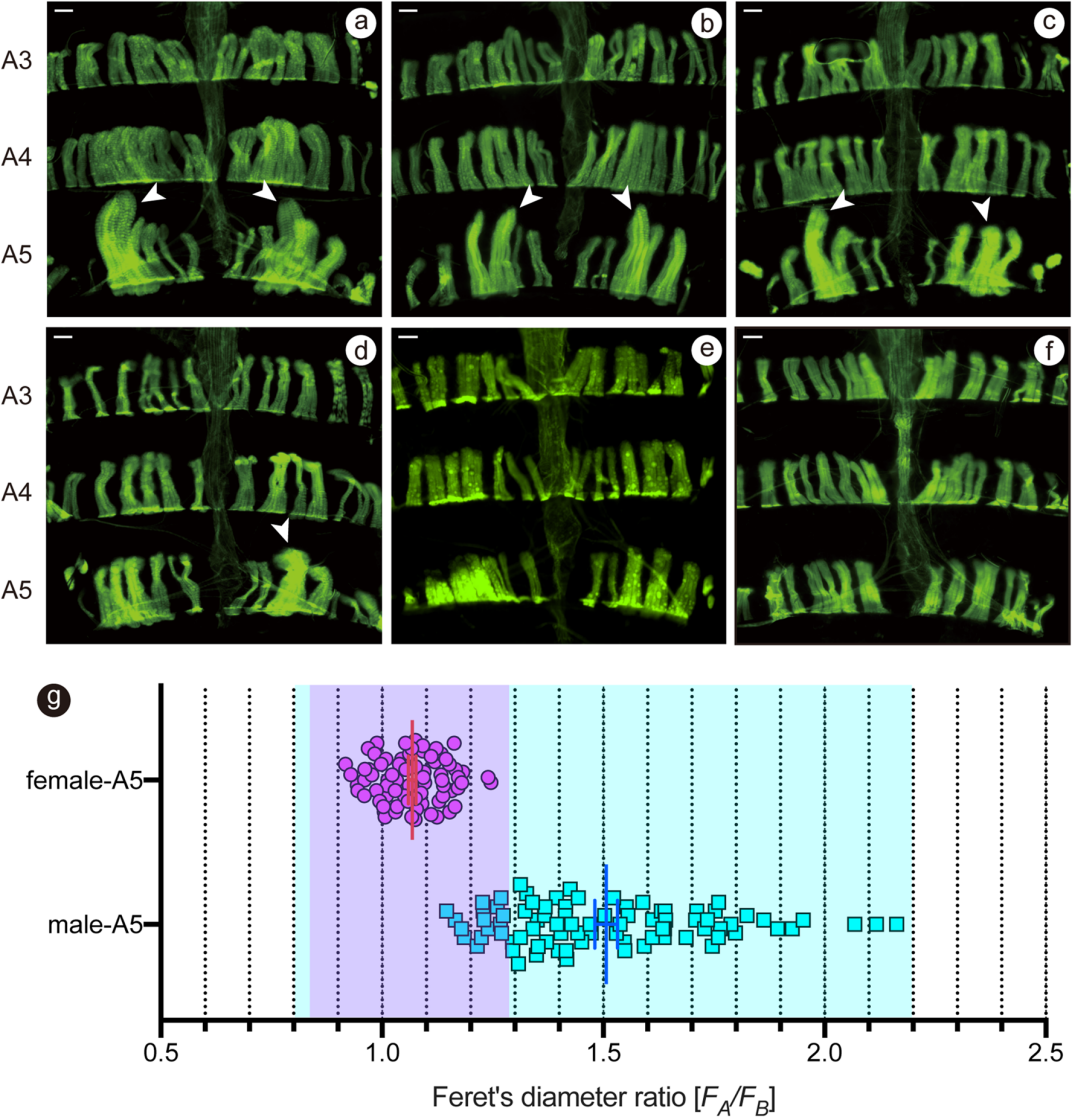
Sexually dimorphic size variations of the largest A5 muscles in *D. kikkawai*. (**a**–**f**) MOL-analogs are visually recognizable bilaterally (**a**–**c**) or unilaterally (**d**) in most males but not females **(f**) and some males (**e**) from A5. (**g**) The scatter plot shows the distribution of mean *F*_*A*_*/F*_*B*_ values as compared between the male and female. Each symbol represents the values estimated for a hemi-segment. The red and blue long lines represent the mean values for female and male values, respectively. The short bar represents the SEM. The blue and pink areas in the graph indicate the 99% confidence intervals (mean ± 3 × SD values) of the muscle size for males and females, respectively.

To determine sexual dimorphisms in the neuromuscular system in the *montium* group flies, we first examined the A5 muscles in *D. kikkawai* (Figs. 2a–f and S3h1–h6). Although the histogram for the male A5 muscles extensively overlapped with that for the female A5 muscles, the male curve (the mean ± SEM of *F*_*A*_*/F*_*B*_: 1.51 ± 0.03) shifted to the right with a larger standard error compared with the female curve (1.07 ± 0.01; Figure S3h6 and Table 1). This sex difference in the *F*_*A*_*/F*_*B*_ distribution was statistically significant (*P* < 0.001; Table 1). To further compare the distribution between the sexes, we plotted all data points for the male and female A5 muscles (Fig. 2g). Strikingly, all values for females were confined within a narrow range (the 99% confidence intervals are defined by colors in Fig. 2g), whereas the values for males were widely distributed, with some overlap with the female data distribution (Fig. 2g). We conclude that *D. kikkawai* exhibits a sexual difference in the formation of a pair of A5 muscles, but this sexual dimorphism forms to a lesser extent when compared with the male-specific MOL development in *D. melanogaster*.

Next, we extended the above analysis to other members of the *montium* group species. We found that 19 out of 41 species examined exhibited significant sex differences in the *F*_*A*_*/F*_*B*_ distribution of A5 muscles: these included *D. baimaii*, *D. barbarae*, *D. birchii*, *D. bocki*, *D. diplacantha*, *D. fengkainensis*, *D. greeni*, *D. kikkawai*, *D. lacteicornis*, *D. leontia*, *D. mayri*, *D. ogumai*, *D. ohnishii*, *D. pectinifera*, *D. serrata*, *D. trapezifrons*, *D. truncata*, *D. tsacasi* and *D. watanabei* (Fig. 1b1–d3 and S3 and Table 1). Among these 19 species, the male A5 muscles with large *F*_*A*_*/F*_*B*_ values equivalent to *F*_*A*_*/F*_*B*_ for the MOL in *D. melanogaster* (1.71 ± 0.03) were found in 2 species, *D. barbarae* (1.60 ± 0.03) and *D. mayri* (1.53 ± 0.02), both of which belong to the *serrata* subgroup (Fig. S3 and Table 1). The male A5 muscles in 5 other species exhibited slightly larger *F*_*A*_*/F*_*B*_ values than that in *D. affinis* (1.34 ± 0.02); the 5 species included *D. baimaii* (1.38 ± 0.02) and *D. lacteicornis* (1.38 ± 0.02) in the *montium* subgroup and *D. bocki* (1.41 ± 0.02), *D. kikkawai* (1.51 ± 0.03) and *D. leontia* (1.40 ± 0.02) in the *kikkawai* subgroup (Figs. 1 and S3). In remaining 12 species, the sex differences in mean *F*_*A*_*/F*_*B*_ values for the largest A5 muscles were less striking yet statistically significant (Table 1 and Figs. 1 and S3).

**Figure 3:**
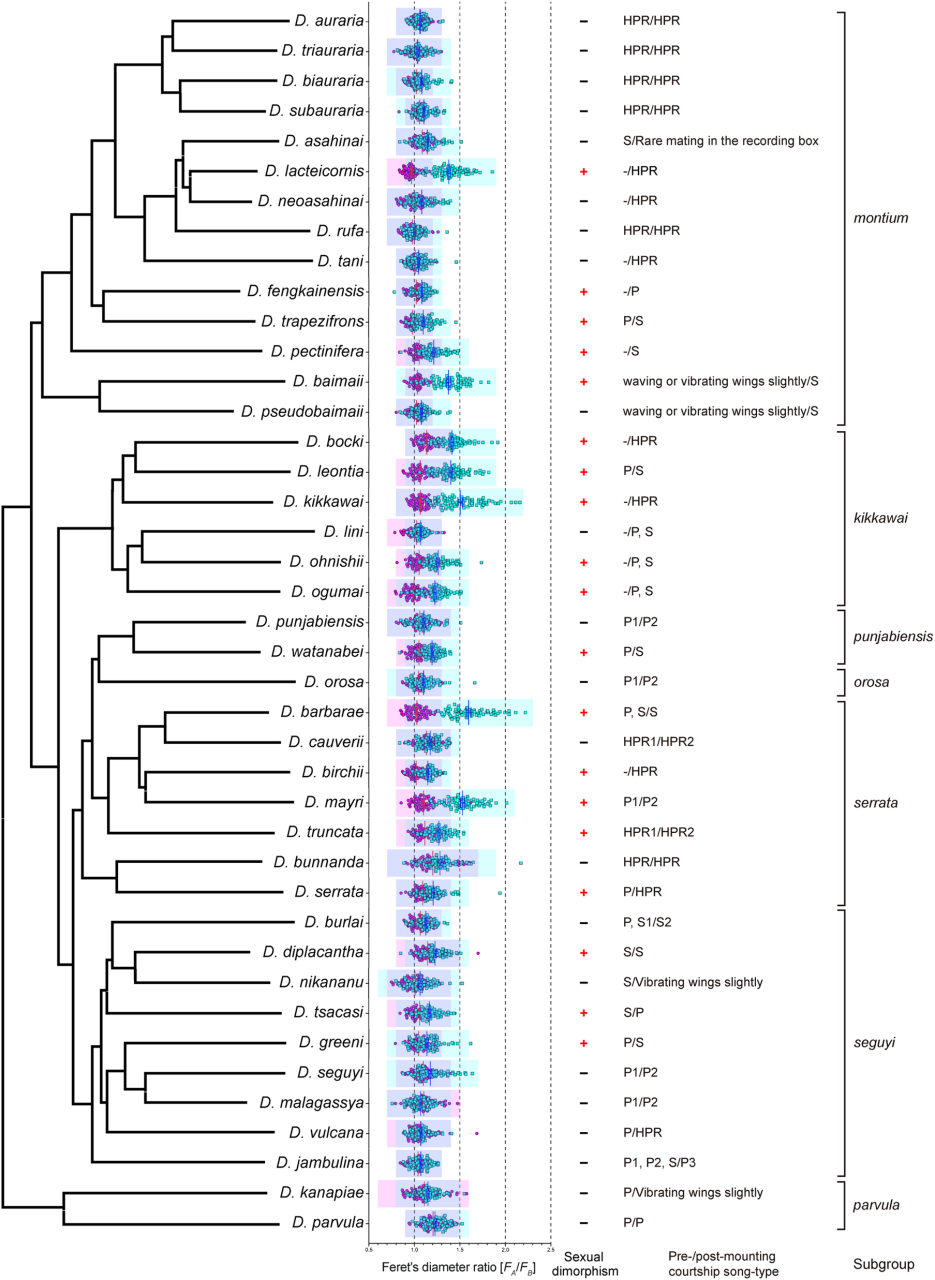
Divergent neuromuscular sexual dimorphisms in A5 among species of the *montium* group. *F*_*A*_*/F*_*B*_ values in the 41 species examined are shown on the phylogenetic tree. The scatter plot shows the distribution of mean *F*_*A*_*/F*_*B*_ values as compared between the male and female. Each symbol represents the values estimated for a hemi-segment. The red and blue long lines represent the mean values for female and male values, respectively. The short bar represents the SEM. The blue and pink areas in the graph indicate the 99% confidence intervals (mean ± 3 × SD values) of the muscle size for males and females, respectively. The neuromuscular system in the A5 segment is sexually dimorphic (+) or sexually monomorphic (−). Courtship song-types reported in our previous paper (Chen et al.^8^) are indicated in the right-hand side of the plots. The song types for pre‐ and post‐mounting songs are shown separated by a hyphen (pre/post): P1–P3, pulse songs (P) distinct in certain song parameters; HPR, high pulse repetition song; S1 and S2, sine songs (S) distinct in certain song parameters; –, no song.

The *F*_*A*_*/F*_*B*_ values for 41 species from the *montium* group are shown as a scatter plot in Fig. 3, which is conjoined with the phylogenetic tree of this fly group. We conclude that some *montium* group species have a MOL analog, the size of which varies markedly within an individual, within a species and/or across species. We note that most of the fly stocks of the *montium* species group used in this study were isofemale lines, and therefore, genetic factors should have contributed only minimally to the observed variations in muscle size within a species. However, we cannot exclude the possibility that the present study underestimates the muscle size variations in wild populations that are genetically heterogeneous.

### Variations in the number of fibers that constitute the MOL analog

As is the case with other muscles, the MOL in *D. melanogaster* is composed of several multinucleate muscle fibers. During development, a single founder myocyte recruits additional myocytes, forming a single fiber^31^. Therefore, the number of nuclei in a fiber corresponds to the total number of myocytes contributed, and the number of fibers coincides with the number of founder myocytes involved in the formation of an MOL. In the *D. melanogaster* species subgroup, the number of fibers contained in an MOL varies within a species and across the species, whereas the total number of myocytes in a fiber is invariant^31^. We therefore asked whether intra-species or inter-species variations in the fiber number exist in the *montium* group and if so, how such variations are correlated with the muscle size or fly phylogeny.

**Figure 4:**
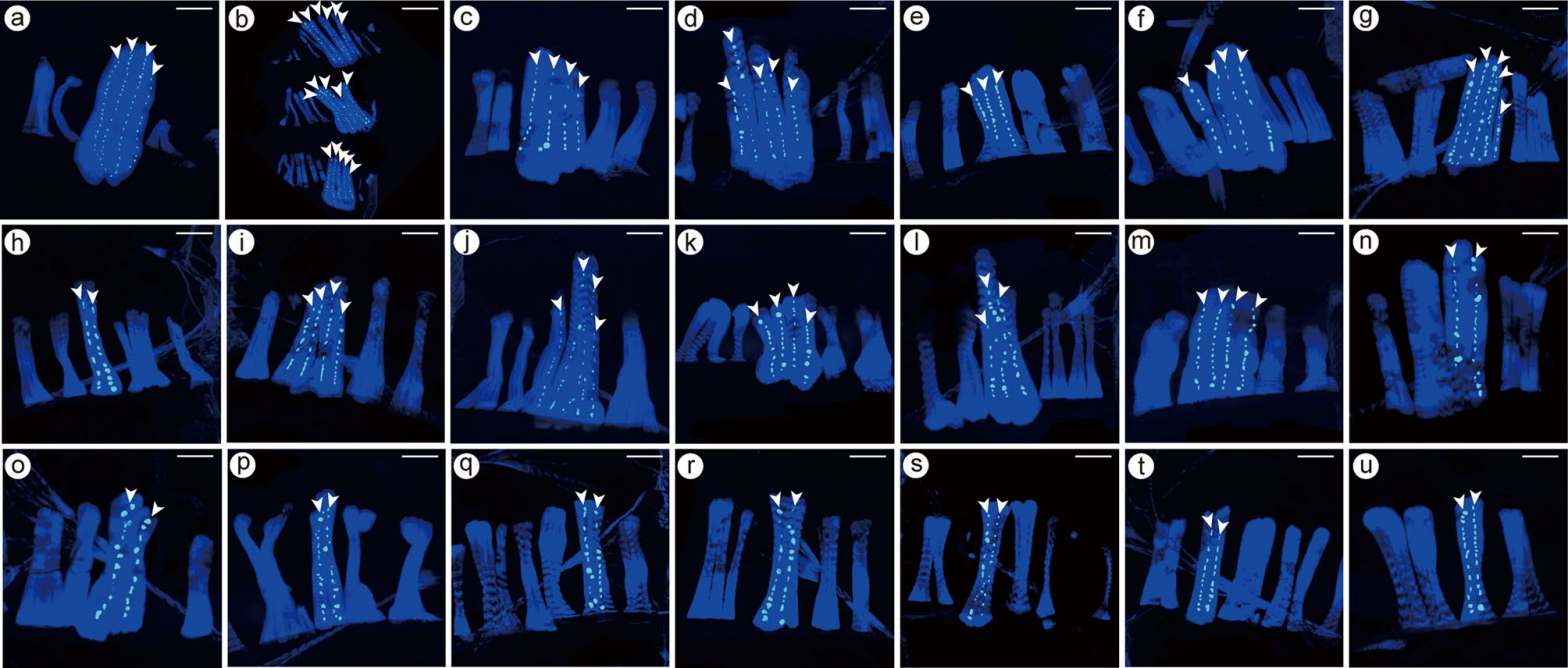
Inter-species variations in the number of fibers composing an MOL or MOL-analog. White dots represent the position of nuclei recorded on a transparency (see Materials and Methods), which aligns along a longitudinal axis, visualizing a single fiber composing the MOL or MOL-analog. Shown are representative examples of MOL nuclear alignments in *D. melanogaster* (**a**), *D. subobscura* (**b**), *D. baimaii* (**c**), *D. barbarae* (**d**), *D. birchii* (**e**), *D. bocki* (**f**), *D. diplacantha* (**g**), *D. fengkainensis*(**h**), *D. greeni* (**i**), *D. kikkawai* (**j**), *D. lacteicornis* (**k**), *D. leontia* (**l**), *D. mayri* (**m**), *D. ogumai* (**n**), *D. ohnishii* (**o**), *D. pectinifera* (**p**), *D. serrata* (**q**), *D. trapezifrons* (**r**), *D. truncata* (**s**), *D. tsacasi* (**t**) and *D. watanabei* (**u**). Scale bars: 50 μm.

Staining for DNA with TO-PRO-3 iodide revealed an array of nuclei along the length of the MOL, and thus visualized the composite muscle fibers, allowing us to determine unequivocally the number of fibers composing an MOL analog (Fig. 4). We performed this analysis in 19 MOL analog-harboring species and 2 outgroup species, *D. melanogaster* and *D. subobscura*. It turned out that all examined species exhibited intra-species variations in the number of fibers composing a MOL analog (Table 2), ranging from 1 to 8 fibers. We also found significant inter-species variations: the largest number was found in *D. barbarae* (mean ± SEM: 5.3 ± 0.5) and the smallest in *D. serrata* (2.1 ± 0.1). However, the number of composite fibers did not appear to be correlated with the entire length of the MOL analog or phylogeny. For example, *D. barbarae* and *D. mayri* belong to the same *serrata* subgroup and have large MOL analogs of similar mean lengths, 1.60 ± 0.03 and 1.53 ± 0.02, respectively (Table 1), yet the MOL analogs of these 2 species are each composed of significantly different numbers of fibers, i.e., 5.3 ± 1.6 and 3.6 ± 0.5 (Table 2). We therefore suggest that the difference in the MOL-analog length across species may not result from a difference in the number of founder cells/composite fibers, which varies from species to species even within the same clade.

**Table 2:** The number of fibers composing an MOL in A5 of male flies of the *Drosophila montium* group species and outgroup species.

Subgroup/(group)	Species	Strain code	*N*	Fiber number		
				Mean ± SEM	Min	Max
Ingroup						
*montium*	*D. baimaii*	ML11023	6	3.1 ± 0.2^bcde^	2	4
	*D. fengkainensis*	XT33	6	2.5 ± 0.3^cde^	2	4
	*D. lacteicornis*	IRUR20	6	3.4 ± 0.4^bcde^	2	6
	*D. pectinifera*	OGS98m	6	2.2 ± 0.2^e^	1	3
	*D. trapezifrons*	Bavi31	6	2.4 ± 0.2^cde^	2	4
*kikkawai*	*D. bocki*	IR2-37	6	3.3 ± 0.3^bcde^	2	5
	*D. kikkawai*	OGH06-01	14	2.9 ± 0.2^bcde^	2	5
	*D. leontia*	AO-2	5	2.7 ± 0.2^cde^	2	3
	*D. ogumai*	RGN3	6	2.3 ± 0.2^cde^	1	4
	*D. ohnishii*	ML45	6	2.7 ± 0.2^cde^	2	4
*punjabiensis*	*D. watanabei*	14028-0531.02	6	2.3 ± 0.2^cde^	3	1
*serrata*	*D. barbarae*	ML11213	5	5.3 ± 0.5^a^	3	7
	*D. birchii*	14028-0521.00	5	2.4 ± 0.2^cde^	2	4
	*D. mayri*	14028-0591.00	6	3.6 ± 0.2^bc^	3	4
	*D. serrata*	Q122	6	2.1 ± 0.1^e^	1	3
	*D. truncata*	RGN179	6	2.7 ± 0.3^cde^	2	5
*seguyi*	*D. diplacantha*	dip05860	6	3.0 ± 0.5^bcde^	1	6
	*D. greeni*	14028-0712.00	6	3.6 ± 0.4^bcd^	2	5
	*D. tsacasi*	14028-0701.00	6	2.2 ± 0.2^de^	1	3
Outgroup						
(*melanogaster*)	*D. melanogaster*	Canton-S	5	4.2 ± 0.2^ab^	3	5
(*obscura*)	*D. subobscura*	zenez	6	5.1 ± 0.2^a^	3	8

*N*: The total number of male flies used in this analysis. The number of fibers (mean ± SEM) and the maximal and minimal number of fibers observed are given in 3 columns on the right-hand side. Statistical differences were evaluated by Kruskal–Wallis test (SPSS 22.0 for Windows) followed by the Tukey’s HSD test, and the results are shown with letters a–e written in superscript following the mean ± SEM values: the same letters indicate that no significant difference was found among the relevant values at *P* ≥ 0.05.

### Is *Act79B* expression a correlate of the MOL?

**Figure 5:**
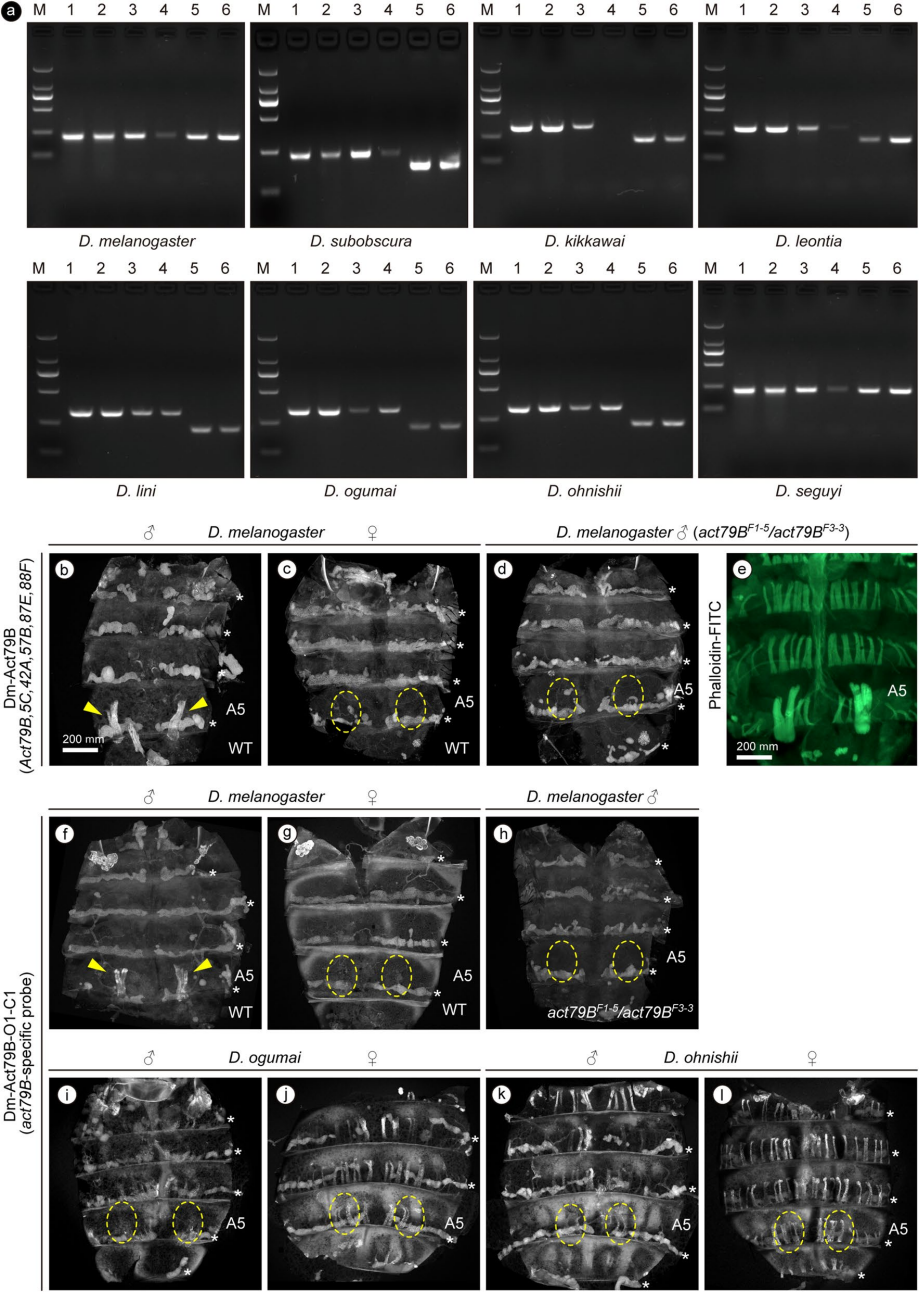
Species differences in sex-biased expression of *Act79B*, an MOL-enriched actin transcript. (**a**) Quantitative RT-PCR analysis. Genomic DNA (lanes 1 and 2) and first-strand cDNA (lanes 3–6) were prepared from tergites of abdominal segments A3–A6 of males (lanes 1, 3 and 5) and females (lanes 2, 4 and 6) of *D. melanogaster*, *D. subobscura*, and 6 species of the *montium* group (indicated below each panel) for *Act79B* (lanes 1–4) and 2 control protein genes, *α-Tubulin* (lane 5) and *Act5C* (lane 6). M: DL2000 DNA marker. Primers used are as shown in Table S3. (**b**–**d,f**–**l**) in situ hybridization analysis with probes for coding (**b**–**d**) or non-coding (**f**–**l**) sequences of the *Act79B* transcript. *Act79B* expression in abdominal muscles in the wild-type (**b,f**) and *Act79B* mutant (**d,h**) males and wild-type females (**c,g**) of *D. melanogaster* and in wild-type males (**i**,**k**) and females (**j**,**l**) of *D. ogumai* (**i,j**) and *D. ohnishii* (**k**,**l**). (**e**) Phalloidin staining reveals the MOL in a *Act79B* mutant male of *D. melanogaster* even though the mutant lacks *Act79B* expression (**d**). The true MOLs with *Act79B* hybridization signals are indicated with arrowheads. The tergite regions typically occupied by the MOL are circled with dotted lines. Oenocytes emit autofluorescence, resulting in a segmentally repeated labelling pattern marked with *. Scale bars: 200 μm (**b**,**e**).

Among the 6 actin genes on the *D. melanogaster* genome, *Act79B* is known to be enriched in the MOL^32,33^. We therefore reasoned that *Act79B* expression may be sexually dimorphic. In keeping with this idea, RT-PCR of RNAs from lysates prepared from tergites showed strongly male-biased expression of *Act79B* in *D. kikkawai* and *D. leontia*, 2 *montium* group members that carry well-developed MOL analogs. Similar male-biased expression was detected in *D. melanogaster* and *D. subobscura*, 2 outgroup species that carry fully developed MOLs (Fig. 5a). Notably, *Act79B* transcript levels were too low to detect in females of *D. kikkawai* and *D. leontia* under our experimental conditions (Fig. 5a). We also measured the *Act79B* mRNA levels in 2 members of the *montium* group that carry smaller MOL analogs, i.e., *D. ogumai* and *D. ohnishii* (both from the *kikkawai* subgroup), as well as 2 members that do not display a neuromuscular sexual dimorphism in the A5 segment, i.e., *D. lini* (from the *kikkawai* subgroup) and *D. seguyi* (from the *seguyi* subgroup). These species exhibited distinct *Act79B* expression patterns: no apparent sex difference in *D. lini*, strongly female-biased expression in *D. ogumai*, moderately female-biased expression in *D. ohnishii* and strongly male-biased expression in *D. seguyi* (Fig. 5a).

To determine the tissue distribution of *Act79B*, in situ hybridization analysis was conducted in tergites of *D. melanogaster*, *D. ogumai* and *D. ohnishii* using the RNA sequence coding for a part of *D. melanogaster Act79B* as a probe (Dm-Act79B probe). We also tested another probe (Dm-Act79B-O1-C1 probe) composed of a 3’UTR sequence of *D. melanogaster Act79B* that is conserved across species for *Act79B* orthologs but not among different actin forms. In *D. melanogaster* tergites, the Dm-Act79B probe yielded signals almost exclusively in the MOL in males and no signals in females under our experimental conditions (Fig. 5b–e). This result was rather unexpected because the sequences of all Actin forms are highly conserved, and the probe used here may cross-react with other Actin proteins. Importantly, *Act79B* mutant males exhibited no discernible hybridization signal in the MOL and other muscles under the same experimental conditions, supporting the notion that the Dm-Act79B probe detected *Act79B* and did not detect other actin mRNAs in the MOL of wild-type *D. melanogaster* males (Fig. 5b–e). A similar result was obtained with the Dm-Act79B-O1-C1 probe which selects for *Act79B* (Fig. 5f–h), suggesting that *Act79B* is primarily expressed in the MOL in *D. melanogaster*. When the Dm-Act79B-O1-C1 probe was used to detect *Act79B* in *D. ogumai* and *D. ohnishii,* hybridization signals were detected in many longitudinal muscles not only in males but also females, including the presumed MOL analogs in males (Fig. 5i–l).

We conclude that, although expression in the MOL is the primary causal factor for male-biased *Act79B* expression in *D. melanogaster* and possibly some other *Drosophila* species with well-developed MOL analogs, male-biased expression of *Act79B* may not be necessarily predictive of the presence of the MOL analogs in the *montium* species group members in which *Act79B* seems to be widely expressed in abdominal muscles irrespective of whether they are MOL analogs or not.

### Ancestral reconstruction of the neuromuscular sexual dimorphism

**Figure 6:**
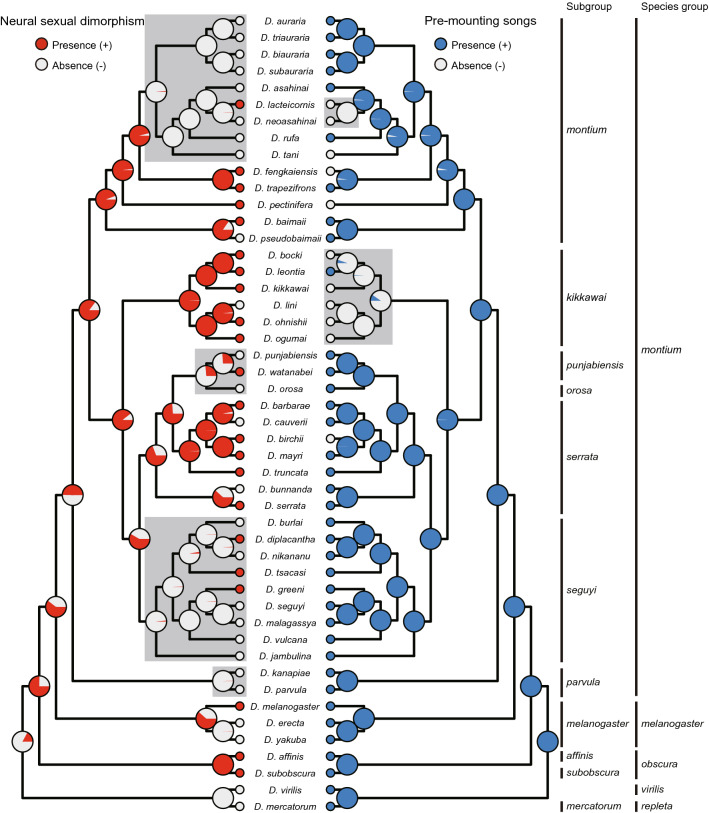
Ancestral reconstruction of the neuromuscular sexual dimorphism in A5 for a Bayesian tree by BBM analysis. The presence or absence of the neuromuscular sexual dimorphism in the A5 segment was determined based on the results shown in Figs. 1 and S3 and Table 1. Pie charts along nodes indicate the probability of ancestral distribution of the neuromuscular sexual dimorphism estimated by BBM analysis (left-hand side panel). It is inferred that the neuromuscular sexual dimorphism was lost at the MRCAs of several lineages indicated by shading. The ancestral distribution of pre-mounting song states (right-hand side panel) is similarly constructed based on our previous observations (summarized in Fig. S5 according to Fig. 3 in Chen et al.^8^) and our unpublished result. The pre-mounting song was probably lost in the two lineages indicated by shading.

The maximum likelihood (ML) and Bayesian analyses generated almost the same tree topology (Figs. S4 and S5), which was largely congruent with those of Chen et al.^8^ and other studies^34,35^. The ancestral state for the presence or absence of a neuromuscular sexual dimorphism in the A5 segment in the subgenus *Sophophora* was reconstructed on the topology of the Bayesian tree (Fig. 6, left-hand side panel). The 19 *montium* group species with the sexually dimorphic neuromuscular system in A5 were scattered across 5 species subgroups, i.e., the *montium* subgroup, the *kikkawai* subgroup, the *punjabiensis* subgroup, the *serrata* subgroup and the *seguyi* subgroup, while in the remaining 2 subgroups, we examined only 1 or 2 species, which did not display the sexual dimorphism. To make the Bayesian tree more comprehensive, we included an additional 7 species outside of the *montium* group, i.e., those from the *melanogaster*, *obscura*, *virilis* and *repleta* species groups (Fig. 6). The reconstruction thus inferred that the most recent common ancestor (MRCA) of *Sophophora* likely carried the MOL. Then, the loss of the neuromuscular sexual dimorphism in the *montium* group would have independently occurred at the MRCAs of the *parvula*, *seguyi*, and *punjabiensis-orosa* subgroups and the *auraria-rufa* species complex (shaded in gray in Fig. 6), although the regain of the neuromuscular sexual dimorphism would have also occurred with the evolution of some species (*D. diplacantha, D. tsacasi, D. greeni, D. watanabei,* and *D. lacteicornis*).

We wanted to determine whether the evolutionary history of the neuromuscular sexual dimorphism may have similarity to that of courtship songs in these species, because both traits are known to be controlled by the same master regulator gene *fruitless* in *D. melanogaster* ^18,19,23^ and *D. subobscura*^26^. The presence or absence of pre- and post-mounting songs in the *montium* group reported by Chen et al.^8^ is summarized in Fig. S5. In Fig. 6 (Fig. 6, right-hand side panel), we included the ancestral reconstruction for the presence or absence of pre-mounting song based on available data^8^, which revealed that pre-mounting song was probably lost in the MRCA of *D. lacteicornis* and *D. neoasahinai* and the *D. kikkawai* species subgroup (shaded in grey; Fig. 6, left-hand side panel). This result suggests that evolutionary changes in the neuromuscular sexual dimorphism in the A5 segment took place independently of those in pre-mounting song production.

## Discussion

In this study, we identified the male-enlarged MOL analogs in 19 out of 41 species of the *montium* species group by rigorous quantification of the muscle size and subsequent statistical tests (Table 1). The MOL analogs found in this phylogenetic branch were much smaller in size than the MOL ortholog characterized in *D. melanogaster* (Tables 1 and S1), and this is probably the reason why Gailey et al.^9^ were unable to detect the MOL analogs in any *montium* group species they examined. 10 out of 12 species examined by Gailey et al.^9^ were evaluated in this study: 4 of them were judged to display the neuromuscular sexual dimorphism in the A5 segment, while the rest were not according to our quantitative criteria. To what extent the MOL analogs are sexually dimorphic varies from species to species (Table 1). *Act79B* mRNA preferentially enriched in the male-specific MOL in *D. melanogaster* appears to be expressed broadly in the abdominal muscles and in both sexes at lower levels in the *montium* group species, judging from our observations in a few select species of this group (Fig. 5). The majority of clades in the *montium* group contain both the species with and those without the neuromuscular sexual dimorphism in the A5 segment, as found in other *Drosophila* groups by Gailey et al.^9^, who explained this mixed configuration by random losses of the MOL in some species within a clade. Our ancestral state reconstitution of the neuromuscular sexual dimorphism favors an alternative view that the neuromuscular sexual dimorphism once present in an old ancestral species was subsequently lost in a few of the most recent common ancestors (MRCAs) of the lineage, and some species regained it thereafter (Fig. 6).

Because the MOL formation is a *fru*-dependent developmental process^20^, we compared the deduced history of the neuromuscular sexual dimorphism in the A5 segment with that of the courtship song patterns, which is another *fru*-dependent process^36^. The ancestral reconstitution suggested that pre-mounting song was lost from two lineages, i.e., the MRCA that delivered both *D. lacteicornis* and *D. neoasahinai* and the MRCA that delivered all *kikkawai* subgroup members (Fig. 6, shaded branches in the pie chart at right). Notably, the former MRCA was likely devoid of the neuromuscular sexual dimorphism in the A5 segment, whereas the latter MRCA probably retained it (Fig. 6, the pie chart at left). In contrast to pre-mounting song, which is limited to only certain species, post-mating song is shared by all *montium* group species thus far examined, precluding the possibility that the presence or absence of post-mating song is correlated with the MOL formation patterns. As Fig. 3 shows, song types were highly divergent from species to species irrespective of whether the species were MOL-sexually dimorphic or MOL-sexually monomorphic. Collectively, the results indicated that no concerted evolutionary changes existed between the neuromuscular sexual dimorphism in the A5 segment and the song pattern, even though both traits were strictly dependent on neural FruM expression. These findings seem to suggest that the neuromuscular sexual dimorphism in the A5 segment and song pattern were shaped under distinct selective pressures, even though both are developmentally controlled by the common master regulator FruM. FruM has been suggested to bind to approximately 130^37^–280^38^ genomic sites, resulting in repression or activation of the transcription of nearby genes^39,40^. It is plausible that FruM turns ON or OFF different sets of target genes in the two developmental contexts, i.e., the neuromuscular sexual dimorphism in the A5 segment and song circuit formation, and that selective pressures acted through distinct downstream genes in each of these cases, leading to the evolutionary paths unique to each trait.

## Materials and methods

### *Drosophila* stocks

The sources of fly stocks are described in Table S2. Flies were maintained on cornmeal-malt medium at 23 °C under a 12:12 h light:dark cycle and 50–60% humidity. Virgin flies were collected and separated into sexes within 12 h of emergence without anaesthesia. Male flies were kept individually in vials (9.5 cm height × 1.5 cm diameter) containing culture medium, whereas females were maintained in vials in groups of five. Reproductively mature virgin flies were used for all experiments: 8-day-old flies for the *montium* group^41^ and 4–6 day-old flies for the outgroup species^30^. No age-dependent change in the MOL size has been reported.

### Tissue dissection, immunohistochemistry and imaging

The experimental procedures for dissection and staining of tissues have been described previously^4^. For observation of the MOL and its analogs, fly abdomens were cut along the dorsal midline and the intestines and other internal tissues were removed to expose the musculature on the internal surface of tergites. The exposed muscles on the tergites were fixed with 4% paraformaldehyde (DF0135; Leagene Biotechnology, China) for 20 min at room temperature (RT), stained with fluorescein isothiocyanate-labeled phalloidin (P5282; Sigma, St. Louis, MO, USA; 1 µg/ml) overnight at 4 °C and examined with a Nikon Eclipse Ti Inverted microscope. For the observation of muscular nuclei, adult dorsal abdomens dissected as above in PBT (0.5% Triton X-100 in PBS) were fixed in 4% paraformaldehyde, washed thoroughly, blocked with PBT + 5% normal goat serum (NGS) for 1 h and incubated with TO-PRO-3 iodide (T3605; Invitrogen, Carlsbad, CA, USA; 1:1000) and fluorescein isothiocyanate labeled phalloidin (P5282; Sigma; 1 µg/ml) overnight at 4 °C. Samples were imaged at 20 × magnification on a Nikon A1 confocal laser microscope. Adobe Photoshop CC software was used to show DNA staining alone in acquired images from the phalloidin-positive regions, so as to clearly visualize the rows of muscular nuclei. The nucleus position was marked on a transparency that overlay a computer screen, on which microscopic images of the MOL or its analogs stained for DNA were displayed.

### Muscle size measurement and statistical analyses

Approximately 40 individuals obtained from several different vials were examined to estimate the size of the abdominal muscles for each genotype or species. The exact numbers of individuals and of hemisegments used are shown in Tables 1 and S1. Both the left and right sib muscles were subjected to size measurements and the two values obtained were treated as independent data, because the bilateral counterparts of a muscle pair form independently from each other^13,14,16^. This treatment unraveled quantitative differences in size between the bilateral counterparts of the MOL analog in single individuals of some *montium* group species.

The size of the MOL analogs and other abdominal muscles was measured by their Feret’s diameter, which is defined as the longest distance between any 2 points along the selection boundary and is also known as the maximum caliper. The Feret’s diameter of muscles was estimated on fluorescent microscopic images of muscles with the aid of the Fiji package of the ImageJ software (https://imagej.net/Welcome). Briefly, the threshold for the fluorescent intensity was set at an appropriate level (typically ~ 14% of the maximal value) to distinguish foreground pixels from background pixels (Fig. S1a) upon choosing an object area to measure with the ROI manager (Fig. S1b). The Fiji program then gave the Feret’s diameter for the object chosen for the measurement (Fig. S1c,d). In every abdominal hemi-segment, Feret’s diameters of the longest muscle as a candidate MOL analog (*F*_*A*_) and the most medial longitudinal muscle as a control (*F*_*B*_) were recorded. The standardized Feret's diameter, *F*_*A*_/*F*_*B*_, was used for the muscle size comparisons between the left and right hemi-segments within the same fly, among conspecific individuals or among the species. *F*_*A*_/*F*_*B*_ can be a value smaller than 1.0 when the most medial longitudinal muscle is the longest muscle in the hemisegment. We chose the most medial muscle as the control because no known MOL and MOL analogs occupy the medial-most position, and no sexual dimorphism is detected in this muscle. Frequency histograms were constructed for the measured *F*_*A*_/*F*_*B*_ for each fly group and fitted by a Gaussian distribution with or without log-transformations. The distributions were further analyzed with GraphPad Prism 8.0 for Mac by one-way ANOVA (the Brown-Forsythe and Welch ANOVA test) followed by the Games-Howell’s comparisons test. In cases where the Gaussian function failed to fit the data, the nonparametric Kruskal–Wallis test was used for statistical comparisons. The confidence interval was set at 99.9% unless otherwise stated. When *F*_*A*_/*F*_*B*_ in males is larger than that in females at the statistically significant level *P* < 0.001, we judge that the males have the sexually dimorphic MOL analog.

### Amplification of 79B actin mRNAs

Total RNA was isolated from the tergite of A3-A5 of a single fly using the TRIzol Reagent and Phasemaker Tubes Complete System (A33251, Invitrogen, USA). cDNA was synthesized using a PrimeScript RT reagent Kit with gDNA Eraser (RR047A, Takara, Japan). All PCR reactions were performed in a 25 μl mixture using Golden Star T6 Super PCR Mix (TSE101, TsingKe, China).

To facilitate the design of the 79B actin specific primers, we downloaded *act79B*, *act5C* and *α-Tub84B* cDNA sequences (Accession Numbers: NT_037436, NC_004354 and NT_033777, respectively) of *D. melanogaster*, and then used the BLAST tool of NCBI to download *act79B* and *act5C* cDNA sequences (Accession Numbers: XM_017167530 and XM_017180678) of *D. kikkawai.* The *D. subobscura* genome assembly^42^ was used for local BLAST via GENETYX-MAC software (version 18.0) to determine the *act79B* and *α-Tub84B* genomic DNA sequences. In particular, it should be noted that since the 5’ ends of *act79B* and *act5C* mRNAs are highly conserved^43^, we need to design primers at the 3’ end to ensure primer specificity. After PCR testing of multiple primer combinations, we selected the primer pairs in Table S3 to amplify the region of *Act79B* and reference gene cDNAs.

### In situ hybridization

RNA scope-based FISH (Advanced Cell diagnostics (ACD), 320850) was performed according to the manufacturer’s protocol with some modifications, using the Dm-Act79B probe (ACD, 451771) or Dm-Act79B-O1-C1 probe to detect *act79B* mRNA. The dorsal muscles, including the MOL, were fixed in 4% PFA for 1 h at 4 °C. A series of MeOH concentrations, i.e., 25%, 50% and 75% in 0.01% PBT (0.01% Tween-20 in PBS), followed by 2 × 100% was used to dehydrate the muscles. After rehydration in 0.01% PBT, the muscles were digested by Protease III for 20 min at RT and post-fixed in 4% PFA for 30 min at 4 °C. The probe hybridization was performed at 40 °C overnight, followed by a second post-fixation in 4% PFA for 10 min at RT. The RNA signal was amplified by Amp 1–4 at 40 °C. After each hybridization step, embryos were washed with 0.02% SSCT (0.02% Tween-20 in 1xSSC). Amp 4 Alt A-FL was used for the fluorescent labeling.

### Phylogenetic analysis

A total of 48 species in the subgenus *Sophophora* of the genus *Drosophila* were used for the phylogenetic analysis, including the 41 species of the *montium* group^8^. Sequences of 2 mitochondrial (*COI* and *COII*) and 3 nuclear (*Adh*, *Amy1*, and *Amyrel*) genes were obtained from GenBank (Table S4). Intron sequences of the nuclear genes were removed before the analysis due to a high degree of alignment ambiguity. Nucleotide sequences of individual gene regions were aligned using MUSCLE^44^ implemented in SeaView 4.7^45^ or MEGA X^46^ with default settings. Individual alignments were concatenated by using FASconCAT 1.0^47^. Phylogenetic trees were constructed based on concatenated sequences, using the maximum likelihood (ML) and Bayesian methods, in which sequences were partitioned according to the best partitioning scheme obtained with PartitionFinder 2.1.1^48^ under the options AICc, “greedy” algorithm, and “models = all”. ML analyses were conducted by using RAxML 8.1.21^49^ with raxmlGUI 1.5beta^50^, in which the “GTRGAMMA” model was applied for all data partitions. A bootstrap analysis of 1000 replicates was performed by using an “ML + rapid bootstrap” search. Bayesian analyses were conducted by using MrBayes 3.2.7a^51^. The best-fit substitution model for each data partition was obtained with PartitionFinder 2.1.1. A Markov-Chain Monte-Carlo (MCMC) search was performed with 4 chains, each of which was run for 10 million generations. Trees were sampled every 100 generations, and the first 25% of the samples were discarded as burn-in. The trace file generated by the Bayesian MCMC runs was inspected in TRACER 1.7.1^52^ to check whether the number of sampling generations and effective sample sizes (ESS) were large enough for reliable parameter estimates.

### Ancestral state reconstruction

Ancestral state reconstruction was carried out for the presence or absence of two characteristics: first, the neuromuscular sexual dimorphism in the A5 segment identified in the present study (Tables 1 and S1), and second, pre-mounting songs based on our previous paper^8^. The reconstruction was performed by using Bayesian binary MCMC (BBM) analysis^53^ implemented in RASP 4.2^54^. Taking phylogenetic uncertainty into account, 10,000 trees randomly selected from 150,000 post burn-in trees generated by MrBayes were used as input trees. The BBM analysis was then run on a consensus Bayesian topology, with the maximum number of areas set to 1 and without allowing null root distribution. The MCMC chain was run for 50,000 generations using 10 chains and sampled every 100 generations. An estimated F81 model^55^ with default Dirichlet distribution (0.5 and 0.5) and equal rates for among-site rate variation was used for the analysis.

## Supplementary Information

Supplementary Figure S1.

Supplementary Figure S2.

Supplementary Figure S3.

Supplementary Figure S4.

Supplementary Figure S5.

Supplementary Information 1.

Supplementary Table S1.

Supplementary Table S2.

Supplementary Table S3.

Supplementary Table S4.

## Data Availability

We agree to deposit our data to a public repository.
