# Supplementary figures and images for: Evolution of a neuromuscular sexual dimorphism in the Drosophila montium species group
Source: Sci Rep. 2021 Jul 27;11:15272. doi: 10.1038/s41598-021-94722-3 (PMC8316392; doi:10.1038/s41598-021-94722-3)

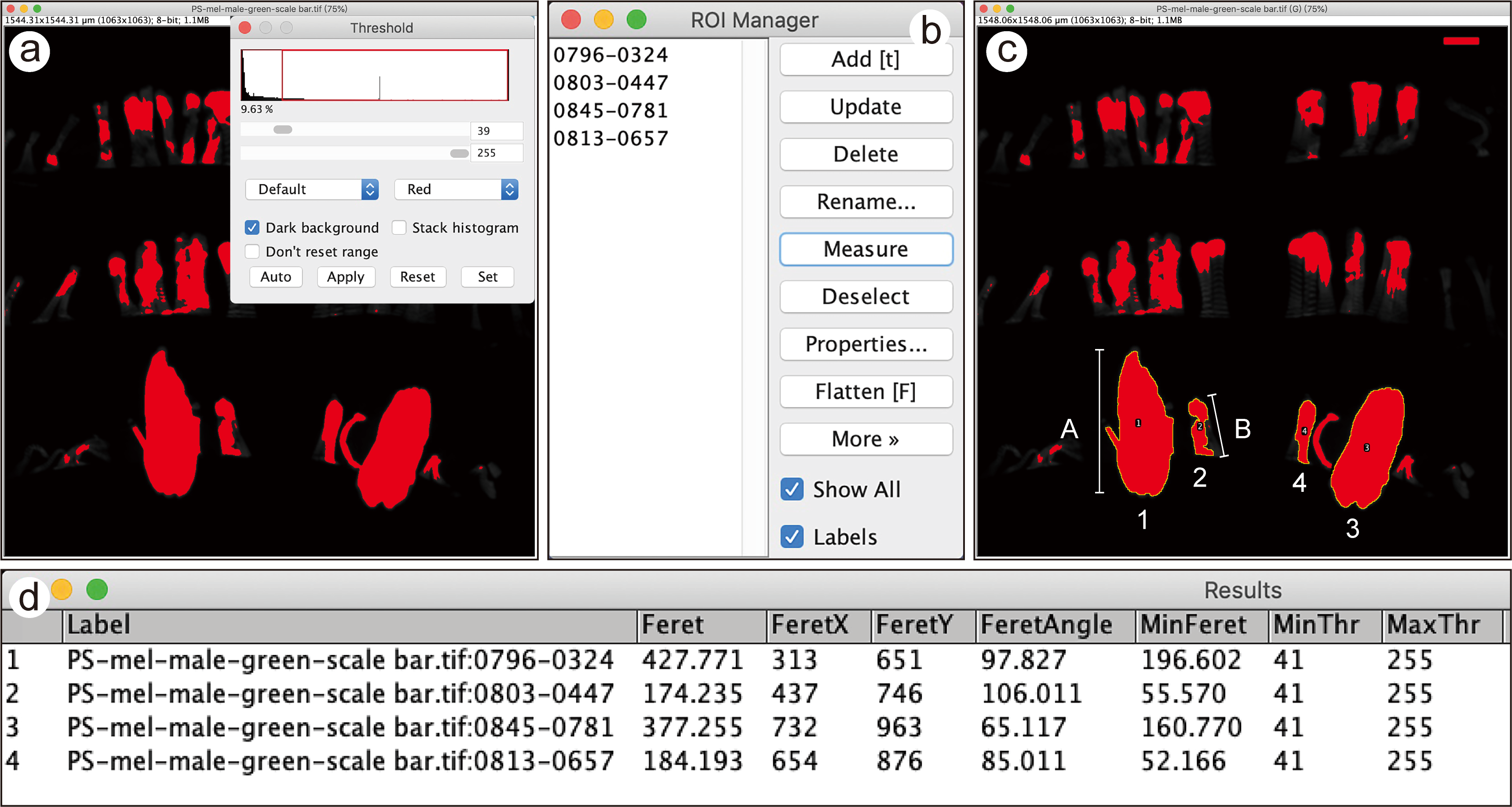

Supplement: Supplementary file 1 — Supplementary Figure S1. [file 41598_2021_94722_MOESM1_ESM.tif]

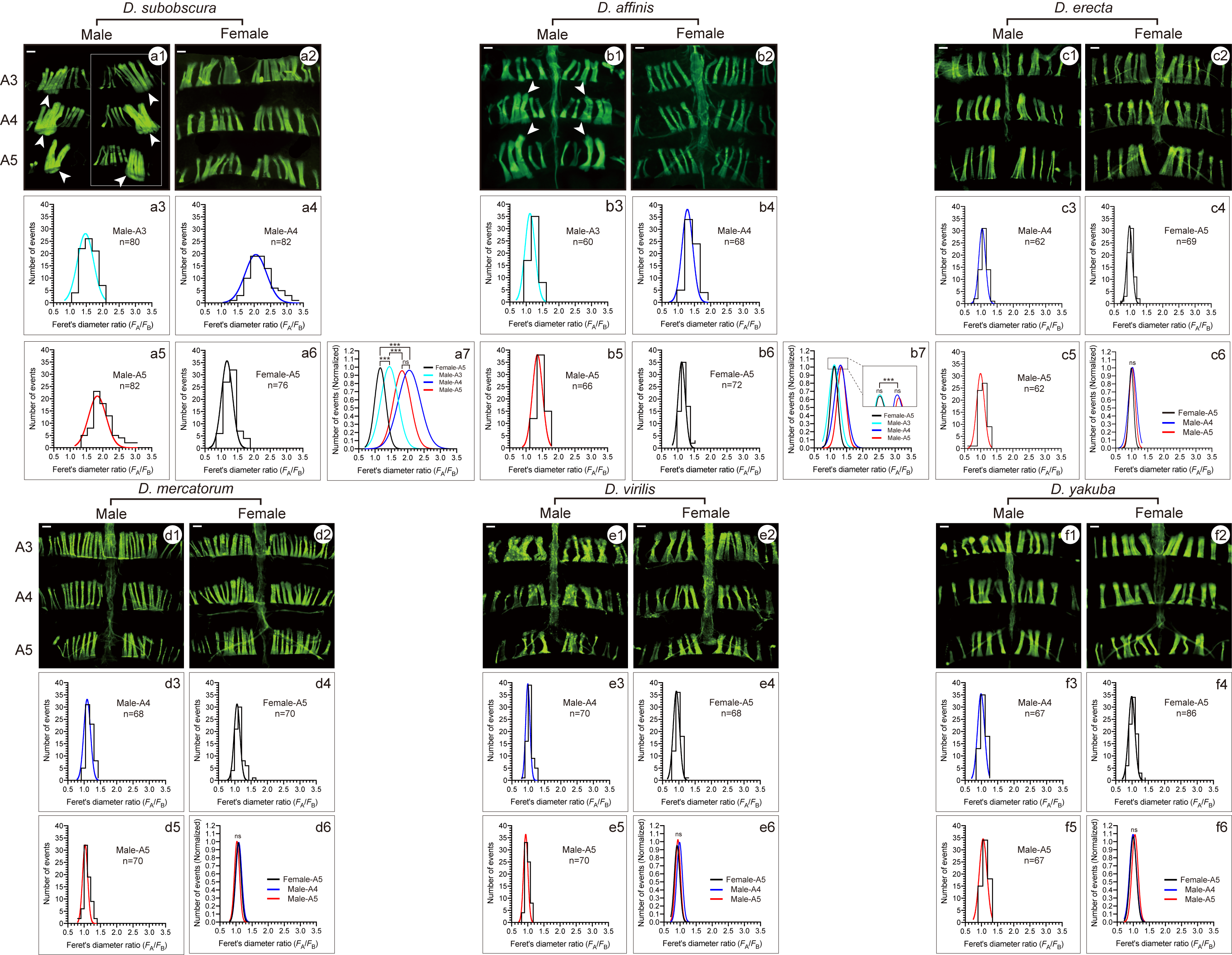

Supplement: Supplementary file 2 — Supplementary Figure S2. [file 41598_2021_94722_MOESM2_ESM.tif]

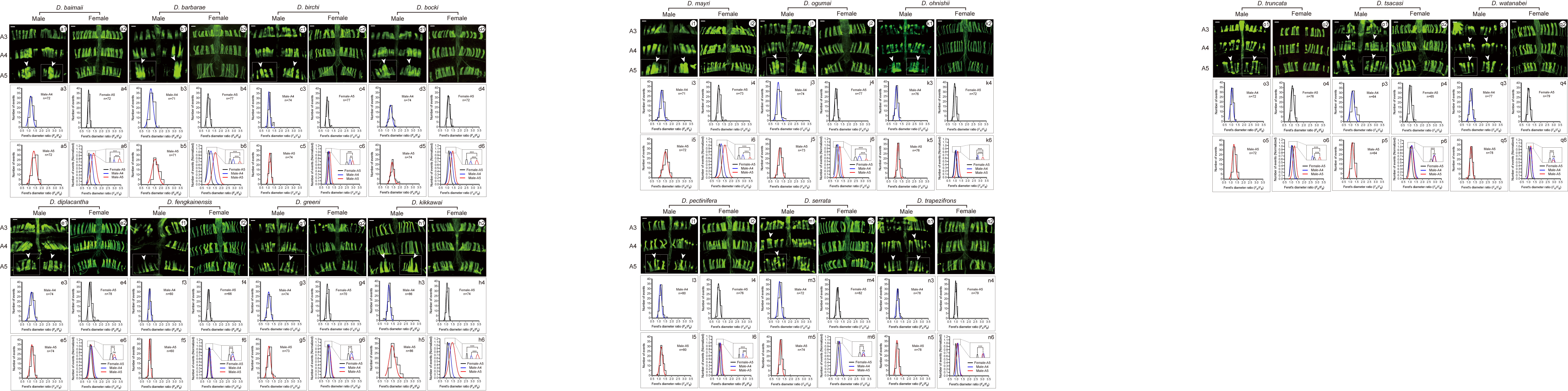

Supplement: Supplementary file 3 — Supplementary Figure S3. [file 41598_2021_94722_MOESM3_ESM.tif]

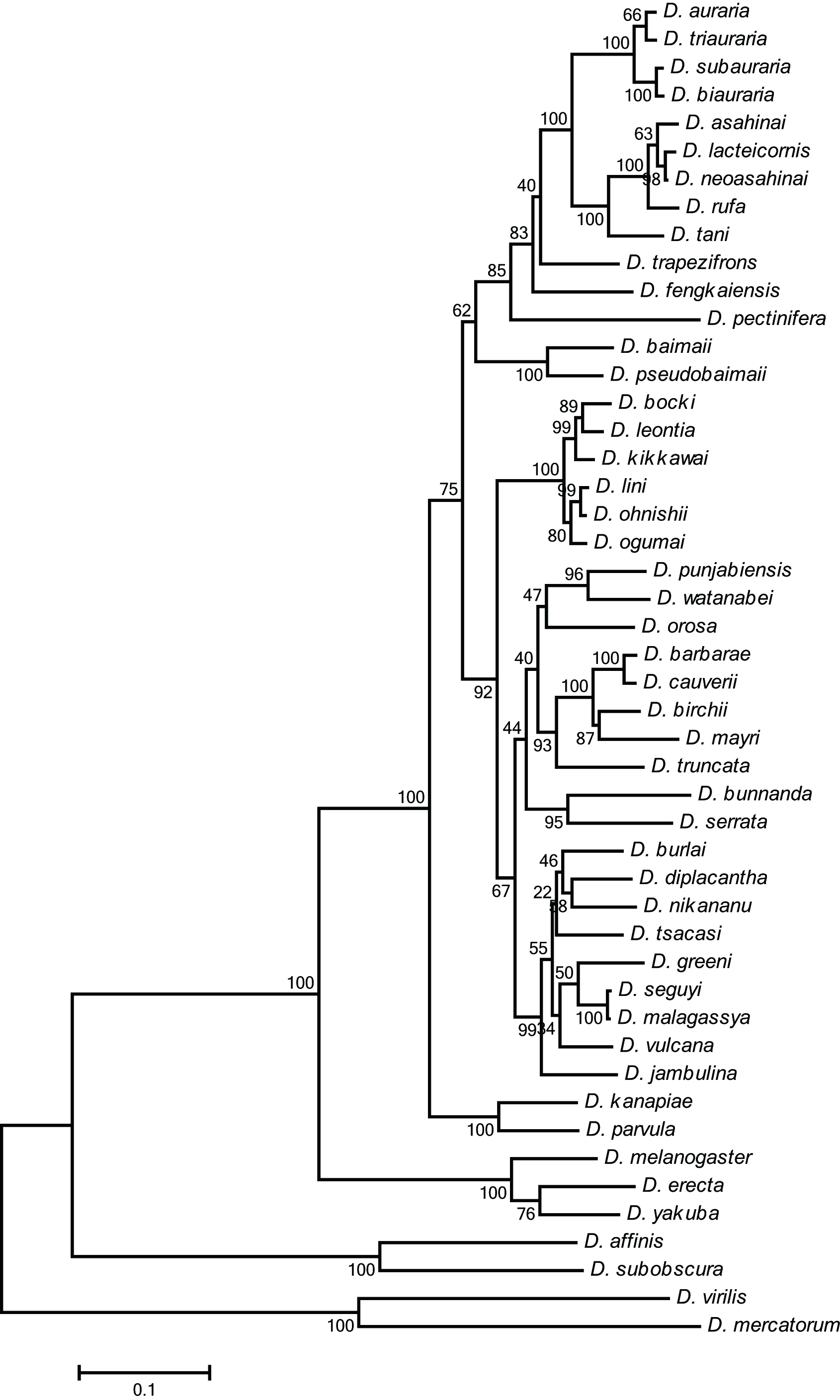

Supplement: Supplementary file 4 — Supplementary Figure S4. [file 41598_2021_94722_MOESM4_ESM.tif]

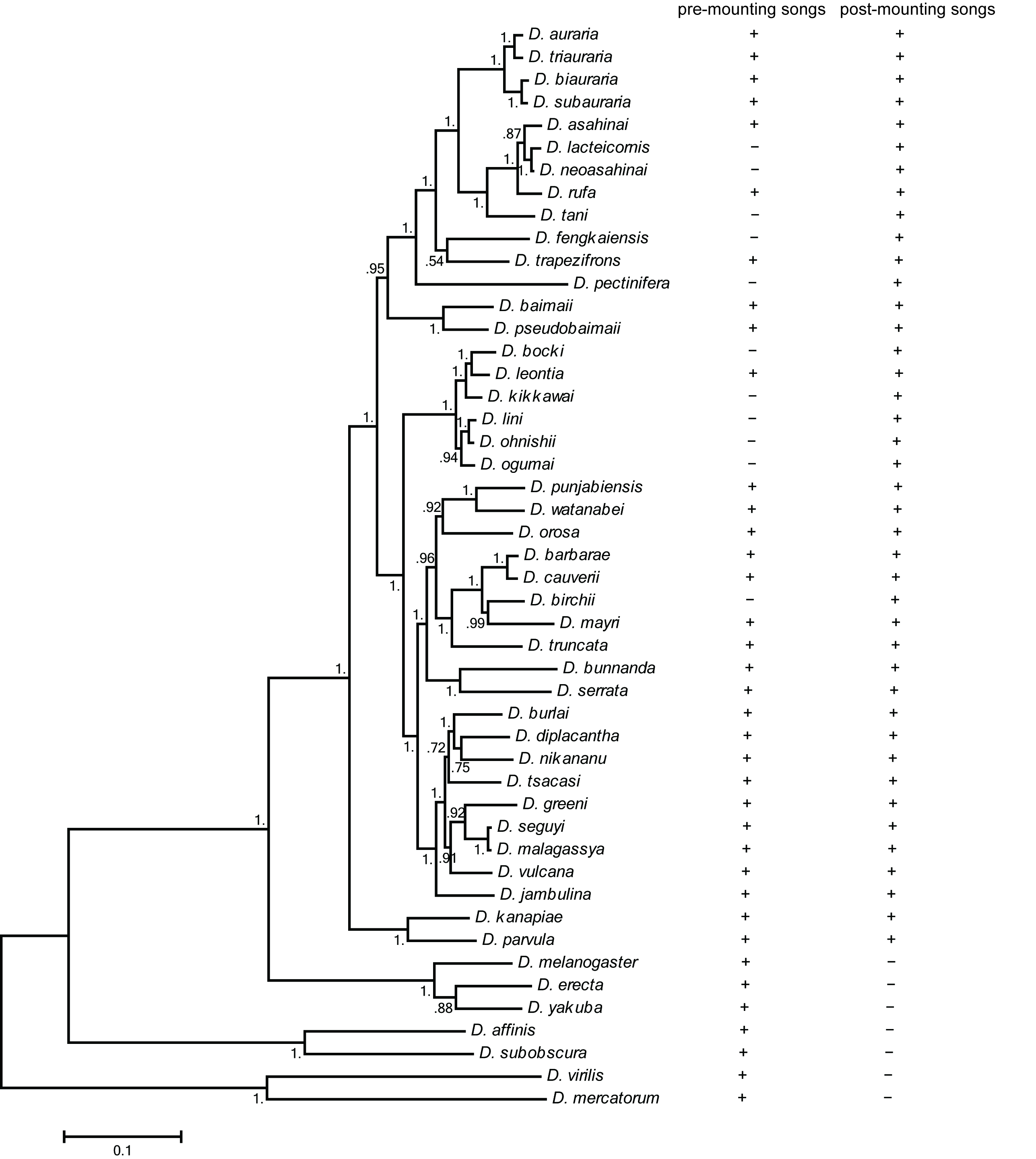

Supplement: Supplementary file 5 — Supplementary Figure S5. [file 41598_2021_94722_MOESM5_ESM.tif]
