# Supplementary material for: Evolution of a neuromuscular sexual dimorphism in the Drosophila montium species group
Source: Sci Rep. 2021 Jul 27;11:15272. doi: 10.1038/s41598-021-94722-3 (PMC8316392; doi:10.1038/s41598-021-94722-3)
Supplement: Supplementary file 6 — Supplementary Information 1. [file 41598_2021_94722_MOESM6_ESM.docx]

**Supplementary figure legends**

**Figure S1. Determination of Feret’s diameter of abdominal muscles with the ImageJ Fiji program.** (**a**) An image of a tergite and associated longitudinal muscles appeared on the computer screen, which also displayed a “Threshold” menu for setting the fluorescent intensity that defines the border between foreground and background pixels. (**b**) The region to be measured in the displayed image is chosen on the “ROI Manager” tab. (**c**, **d**) Bilateral pairs of the MOL (labeled as 1 and 3) and most-medial conventional muscle (labeled as 2 and 4) subjected to measurements are indicated on the display (**c**) and the Feret’s diameters and other measured values are shown in the “Results” window (**d**). The ratio of the Feret’s diameter (*F_A_/F_B_*) of the MOL (A) relative to that of the conventional muscle (B) is used for muscle size comparisons.

**Figure S2. Characteristics of MOL analogs in two outgroup species.** (**a1**, **a2**, **b1**, **b2**) Examples of the abdominal musculatures in male (**a1**, **b1**) and female (**a2**, **b2**) flies of *D. subobscura* (**a1**, **a2**), *D. affinis* (**b1**, **b2**), *D. erecta* (**c1**, **c2**), *D. mercatorum* (**d1**, **d2**), *D. virilis* (**e1**, **e2**) and *D. yakuba* (**f1**, **f2**). Scale bar: 100 µm. Presumptive MOL analogs are indicated with arrowheads. (**a3**–**a7**, **b3**–**b7, c3**–**c6, d3**–**d6, e3**–**e6, f3**–**f6**) Frequency histograms of *F_A_/F_B_* for the largest muscles in male A3 (**a3**, **b3**), male A4 (**a4**, **b4, c3, d3, e3, f3**), male A5 (**a5**, **b5, c5, d5, e5, f5**) and female A5 (**a6**, **b6, c4, d4, e4, f4**) in *D. subobscura* (**a3**–**a7**), *D. affinis* (**b3**–**b7**), *D. erecta* (**c3**–**c6**), *D. mercatorum* (**d3**–**d6**), *D. virilis* (**e3**–**e6**) and *D. yakuba* (**f3**–**f6**). The histograms were fitted with Gaussian distributions and fitted curves (male-A3: cyan; male-A4: blue; male-A5: red; female-A5: black) were displayed on the same coordinate axes for each species to facilitate comparisons. Statistical differences were evaluated by one-way ANOVA (the Brown-Forsythe and Welch ANOVA test) followed by the Games-Howell’s comparisons test; ***P＜0.001, **0.001≤P＜0.01, *0.01≤P＜0.05, ns: not significant, P≥0.05.

**Figure S3. Quantitative analyses of MOL analogs in the *montium* group.** Representative images of dorsal abdominal muscles of male (**a1**–**q1**) and female (**a2**–**q2**) flies of the indicated species of the *montium* group. The MOL and its analogs are indicated with arrowheads and the regions shown in Figure 4 to visualize nuclei are boxed. Scale bar: 100 µm. The curves in the 6th panels of (**a**) to (**q**) compare *F_A_/F_B_* distributions for the male A5 (red lines), female (black lines) and male A4 (blue lines). The statistical differences were evaluated by the Brown-Forsythe and Welch ANOVA or Kruskal-Wallis test; ***P＜0.001, **0.001≤P＜0.01, ns: not significant, P≥0.05.

**Figure S4. A maximum likelihood tree of select *Drosophila* species.** The phylogeny was deduced based on 2 mitochondrial (*COI* and *COII*) and 3 nuclear (*Adh*, *Amy1* and *Amyrel*) genes for 41 species of the *montium* group and 7 outgroup species in the subgenus *Sophophora*. Branch support is indicated by the bootstrap value.

**Figure S5. A Bayesian phylogenetic tree of select *Drosophila* species with presence or absence of pre-mounting songs.** The phylogeny was deduced based on 2 mitochondrial (*COI* and *COII*) and 3 nuclear (*Adh*, *Amy1* and *Amyrel*) genes for 41 species of the *Drosophila montium* group and 7 outgroup species in the subgenus *Sophophora*. Branch support is indicated by the Bayesian posterior probability (≥ 0.9). The presence (+) or absence (−) of pre-mounting and post-mounting songs is indicated aside the Bayesian phylogenetic tree according to Figure 3 of our previous paper (Chen et al., 2019) and unpublished data.
